# Random forest-based imputation outperforms other methods for imputing LC-MS metabolomics data: a comparative study

**DOI:** 10.1186/s12859-019-3110-0

**Published:** 2019-10-11

**Authors:** Marietta Kokla, Jyrki Virtanen, Marjukka Kolehmainen, Jussi Paananen, Kati Hanhineva

**Affiliations:** 1 Institute of Public Health and Clinical Nutrition, University of Eastern Finland, Kuopio Campus, P.O. Box 1627, FI-70211 Kuopio, Finland; 2 VTT Technical Research Centre of Finland Ltd, P.O. Box 1000, FI-02044 VTT Espoo, Finland; 3 Institute of Biomedicine, University of Eastern Finland, Kuopio Campus, P.O. Box 1627, FI-70211 Kuopio, Finland

**Keywords:** Metabolomics, Imputation, Missing values, High dimensional data, RF, MAR, MNAR, MCAR

## Abstract

**Background:**

LC-MS technology makes it possible to measure the relative abundance of numerous molecular features of a sample in single analysis. However, especially non-targeted metabolite profiling approaches generate vast arrays of data that are prone to aberrations such as missing values. No matter the reason for the missing values in the data, coherent and complete data matrix is always a pre-requisite for accurate and reliable statistical analysis. Therefore, there is a need for proper imputation strategies that account for the missingness and reduce the bias in the statistical analysis.

**Results:**

Here we present our results after evaluating nine imputation methods in four different percentages of missing values of different origin. The performance of each imputation method was analyzed by Normalized Root Mean Squared Error (NRMSE). We demonstrated that random forest (RF) had the lowest NRMSE in the estimation of missing values for Missing at Random (MAR) and Missing Completely at Random (MCAR). In case of absent values due to Missing Not at Random (MNAR), the left truncated data was best imputed with minimum value imputation. We also tested the different imputation methods for datasets containing missing data of various origin, and RF was the most accurate method in all cases. The results were obtained by repeating the evaluation process 100 times with the use of metabolomics datasets where the missing values were introduced to represent absent data of different origin.

**Conclusion:**

Type and rate of missingness affects the performance and suitability of imputation methods. RF-based imputation method performs best in most of the tested scenarios, including combinations of different types and rates of missingness. Therefore, we recommend using random forest-based imputation for imputing missing metabolomics data, and especially in situations where the types of missingness are not known in advance.

## Background

Metabolomics studies involve investigation of small molecules or molecular features, which are end results of cellular metabolism in biological samples. The primary motivation behind the technology is to profile as many molecular features as possible with the chosen instrumental set-up and thereafter study e.g. alterations in metabolism under different conditions. One of the most commonly used technologies in metabolomics is liquid chromatography combined to mass spectrometry (LC-MS) which generates a vast amount of multivariate data amenable for multiple biostatistical, bioinformatical and any other computational data-analytical approaches [1, 2].

A pre-requisite for reliable data-analysis in metabolomics experiment is that the quality of the data is monitored efficiently. One of the main drawbacks in LC-MS metabolomics data is that it typically may contain a large proportion of missing values, even in the range of 30–50% [3, 4]. The procedure of treating missing values is called imputation, and it focuses on replacing the missing data with values using the information that is available from the existing data. So far, several methods have been introduced for the imputation of metabolomics data [3, 5–7].

The missingness of data is generally due to either true absence of the compound in the measured sample, or the molecular feature can be present in the sample at a concentration below the detection limit of the mass spectrometer. However, in many cases the missing data value results from inappropriate transformation of the measured MS signal to the numerical data format. The raw data processing involves various steps including peak detection, peak alignment, adduct/neutral loss detection, baseline correction and noise reduction. Therefore it is one of the most challenging computational processes in the metabolomics experiment and thus prone to errors [8]. Thus, the related statistical analysis and the interpretation of metabolomics data will be biased, in case the missing values are not treated properly.

Given the magnitude of missing data and the ambiguity that surrounds the statistical analysis of metabolomics datasets, it is desirable first to investigate and then consider the properties of missing data prior to applying any imputation methods. Rubin and Little have established the foundations of missing data theory [9, 10] and according to them missing data can be classified into three missing mechanisms based on the nature of the absence of values from the data matrix: Missing Completely at Random (MCAR), Missing at Random (MAR) and Missing Not at Random (MNAR). The first mechanism, MCAR, describes the process that the missing values cannot be attributed neither to the molecular features that are present or to the molecular features that are missing in the samples. This mechanism is characterized by a randomness in the occurrence of missing data, which could also be accounted as a zero correlation between the missing and the observed part in the data. In the MAR mechanism, we observe a systematic correlation between the missing molecular features and the observed data, but not with the missing data itself, meaning that the probability of a molecular feature being missing is determined by other observed molecular features. Lastly, MNAR is the most troublesome mechanism, because it depends on the unobserved part of the data, when missing values are neither MCAR nor MAR. MNAR denotes the process that the value of the molecular feature is causal for missingness and that is why it is missing, and this type of missing data is usually characterized in many metabolomics studies as left truncated data (molecular features occur below the detection limit) [11].

Furthermore, diagnosing what type of mechanisms of missingness there are in a dataset is challenging, and there is not yet a solid straightforward method to diagnose these mechanisms [12, 13]. Many studies have compared imputation strategies such as different variations of K-nearest neighbors or used machine learning techniques to replace missing values, and investigated how they can alter the biological information within simulated or real metabolomics datasets [2, 5, 6, 11, 14–18].

In order to obtain reliable metabolomics data, it is necessary to find the most suitable method or approach for treating the missing values of various origin. Hence, we performed a detailed evaluation on the performance of various imputation methods on sub-sets of data generated from human plasma samples measured with LC-MS based non-targeted metabolite profiling analysis. In this work, missing data was simulated with the three main missing mechanisms (MCAR, MAR, MNAR) and four additional combined mechanisms (MCAR-MAR, MCAR-MNAR, MAR-MNAR, MCAR-MAR-MNAR) which we created in order to introduce a more realistic missing data structures. The datasets containing missing values based on the above seven different missing mechanisms were imputed with nine commonly applied imputation methods in metabolomics: ZERO, MEAN, minimum value (MIN), half minimum (½ MIN), Singular Value Decomposition (SVD), Probabilistic Principal Component Analysis (PPCA), Bayesian Principal Component Analysis (BPCA), Random Forest (RF), and K-Nearest Neighbors (KNN).

## Results

**Fig. 1:**
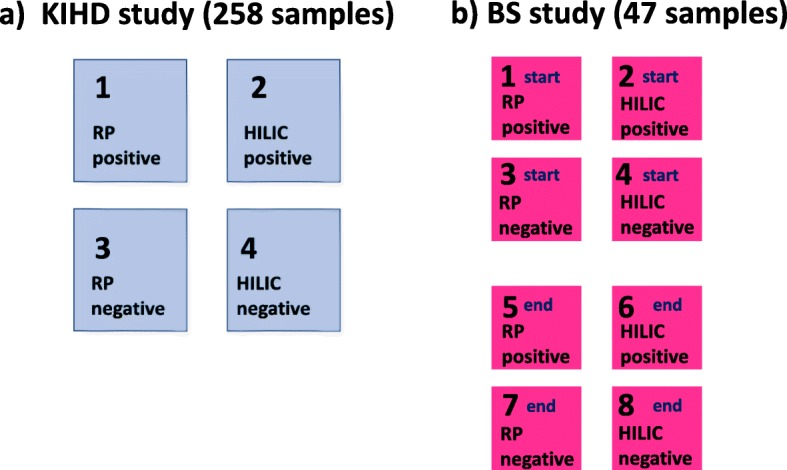
Description of the datasets. Datasets from two different studies were used; KIHD (Kuopio Ischaemic Heart Disease Risk Factor Study) and BS (Berry Study).The data were analyzed with LC-MS technology and each analytical chromatographic mode is consider a separate dataset. In total 12 datasets were used **a** four datasets from the KIHD study and **b** eight datasets from the BS intervention study (four chromatographic modes per time point)

**Fig. 2:**
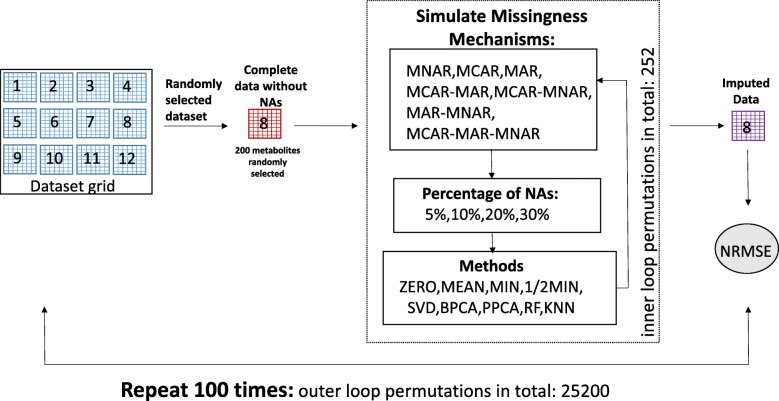
Imputation work flow. In every dataset from the dataset grid (12 datasets), which is randomly selected, the missing values are filtered out and 200 molecular features are randomly chosen. Then seven different missing mechanisms are simulated; Missing Completely At Random (MCAR), Missing At Random (MAR), Missing Not At Random (MNAR), MCAR-MAR, MCAR-MNAR, MNAR-MAR, MNAR-MCAR-MAR, in four different percentages (5, 10, 20, and 30%) of missing data. In every dataset that is chosen randomly, nine imputation are used in order to investigate the performance of the imputation methods in estimating missing values. The evaluation of the methods is done using NRMSE. The whole processes are repeated 100 times

In order to test the performance of the nine different imputation methods, we generated sub-datasets from 12 LC-MS metabolomics datasets (Fig. 1), and simulated missing values to these according to seven different missing mechanisms; MCAR, MAR, MNAR, MCAR-MAR, MCAR-MNAR, MAR-MNAR, and MCAR-MAR-MNAR, in four different proportions of missing values (5, 10, 20 and 30%). The imputation process was repeated 100 times as visualized in the workflow chart in Fig. 2. Furthermore, in order to avoid potentially biased comparisons, we have explored the performance of KNN method by optimizing the parameter settings to reach optimal performance. The number of neighbors K was chosen to be equal with 10. In the RF, method there is no need for tuning the parameters nor does it require assumptions about distributional aspects of the data as suggested [19] so we chose the default values of the missForest function; maximum iterations was set to 10 and the number of trees was chosen to be 100.

**Fig. 3:**
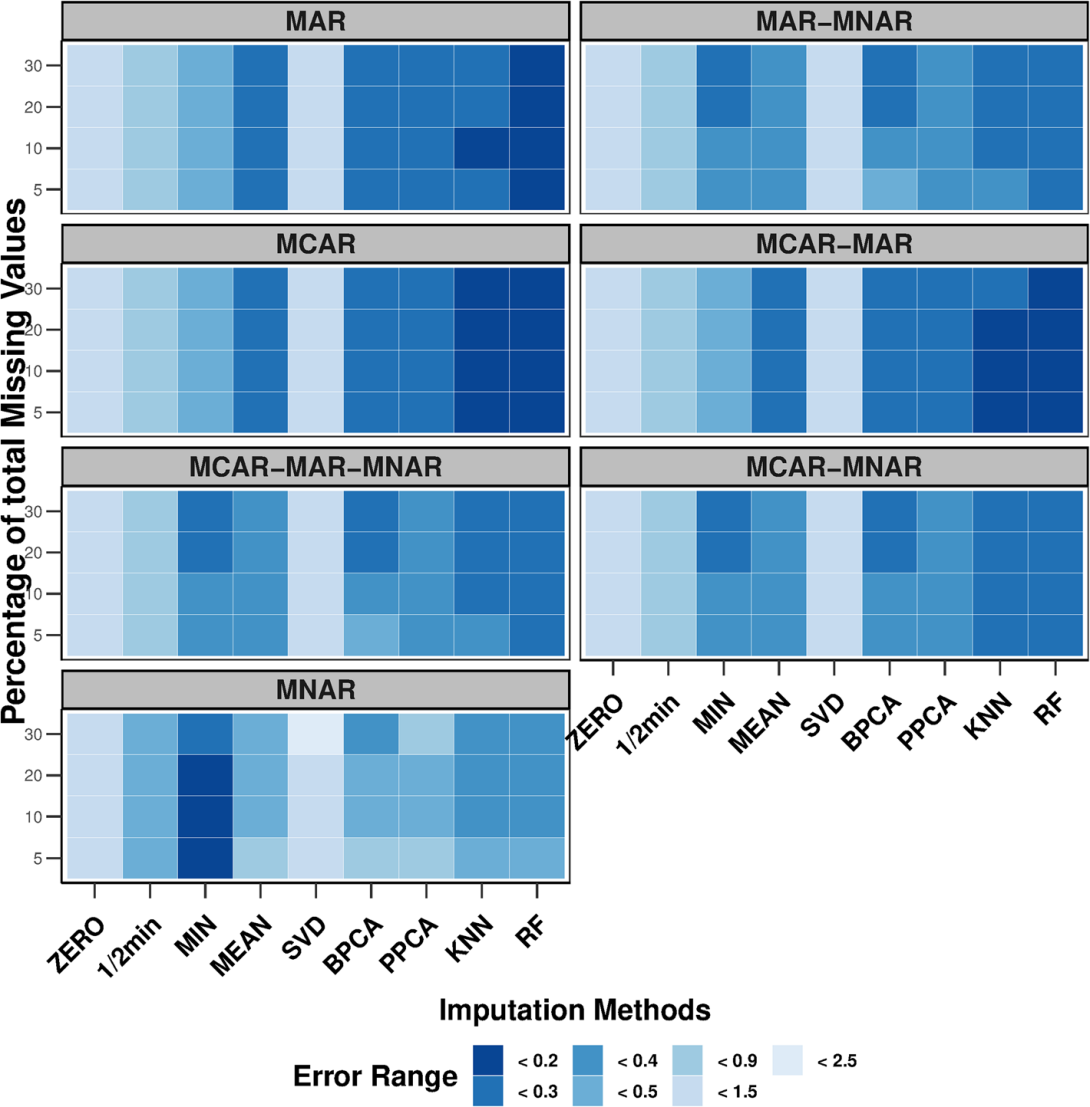
Heatmaps. Heat maps representing the average performance of nine imputation methods after 100 permutations; zero (ZERO), ½ -minimum (½ MIN), minimum (MIN), Random forest (RF), mean (MEAN), K-nearest neighbor (KNN), Bayesian Principal Component Analysis (BPCA), Probabilistic Principal Component Analysis (PPCA), Singular Value Decomposition (SVD) in seven missing mechanisms (each box); MCAR, MAR, MNAR, MCAR-MAR, MCAR-MNAR, MAR-MNAR, MCAR-MAR-MNAR. The darker blue color indicates that the error is small whereas, when the color transforms to lighter shades, this is an indication that the error becomes higher by the corresponding imputation method

The tested imputation methods were ZERO, MEAN, MIN, ½ MIN, SVD, PPCA, BPCA, RF, and KNN. Results from the performances of all the imputation methods are illustrated in Fig. 3 as heatmaps. The heatmaps show the average Normalized Root Mean Square Error (NRMSE), which calculates the difference in the estimation between the imputed value and the original value for every molecular feature that contains missing values, after 100 permutations. Each heatmap reflects one missing mechanism and the different shades of blue are associated with the different values of NRMSE that indicate the performance of each imputation method in four different percentages of missing data.

### Comparison of imputation methods based on heatmaps

In the MCAR case, RF and KNN performed the best by having the smallest NRMSEs, while PPCA, BPCA and MEAN, even though having similar performances with each other, could not outperform RF and KNN. SVD was the method with the highest NRMSEs compared to the other methods. ZERO imputation, ½ MIN and MIN method had smaller NRMSEs compared to the SVD but still higher than RF and KNN.

The MAR missing mechanism had relatively similar results as the MCAR missing mechanism, with RF being the method with significantly lower NRMSEs than the others. The NRMSEs of the KNN were higher than the ones coming from RF when 5% of the data were missing. When 10% of the data were missing, KNN performs the same as RF, however, for higher percentages of missing values the error increased again. In the case of the other imputation methods, the trend of the performances were similar to the results obtained for the MCAR case.

In the MNAR missing mechanism, MIN was the best performing method compared to the other ones. It is interesting to note that when the number of missing values increases and starts reaching 30%, the NRMSE of the MIN also increases. RF and KNN had similar results with each other and achieved their best smallest NRMSEs between 10 to 30% proportion of the missing values. ZERO and SVD were consistent in having high errors for all the percentages of missing values compared to the other methods. Furthermore, MIN was more robust than ½ MIN with smaller NRMSEs for all the percentages of missing values. PPCA, BPCA and MEAN started with relative high NRMSEs when 5% of the data were missing compared to the MIN imputation, but the NRMSEs decreased, although not significantly, when the range of missingness was between 10 to 20%. When the missingness reached 30%, the NRMSE of BPCA and MEAN continued to drop but for PPCA method it increased again.

For the mixed missingness MCAR-MAR, RF and KNN had the smallest NRMSE. RF’s NRMSE was constant for all the percentages of missing values in comparison with KNN, errors of which increased when the range of missing values reached 30%. BPCA, PPCA, and MEAN had similar results for all the four percentages of missing values. ZERO and SVD had similar performances with high NRMSEs in all percentages. Between MIN and ½ MIN methods, MIN value had the smallest NRMSEs but still higher than RF, KNN, BPCA, PPCA and MEAN.

With MAR-MNAR, RF performed the best in all the missing percentages. KNN performed as good as RF, with a small increase of the error when 5% of the data were missing. MIN together with BPCA had similar performances with KNN and RF, when the percentages of missing values were between 20 and 30%, but for smaller percentages of missing values (between 5 and 10%) they had relatively high NRMSEs compared to RF. The rest of the methods as an overall performance had higher NRMSEs compare to RF.

By combining MCAR and MNAR mechanisms together (MCAR-MNAR) we had similar performances in the imputation methods as in the MAR-MNAR case. Once again KNN and RF were the ones with the smallest errors. The only difference was that at the 5% range of missing values, KNN and BPCA had smaller NRMSEs compared to the MAR-MNAR case.

Lastly, when we had three missing value mechanisms mixed together (MCAR-MAR-MNAR) the results looked similar as with the MAR-MNAR missing mechanism. The best performing method was RF followed by KNN.

### Summary results

**Fig. 4:**
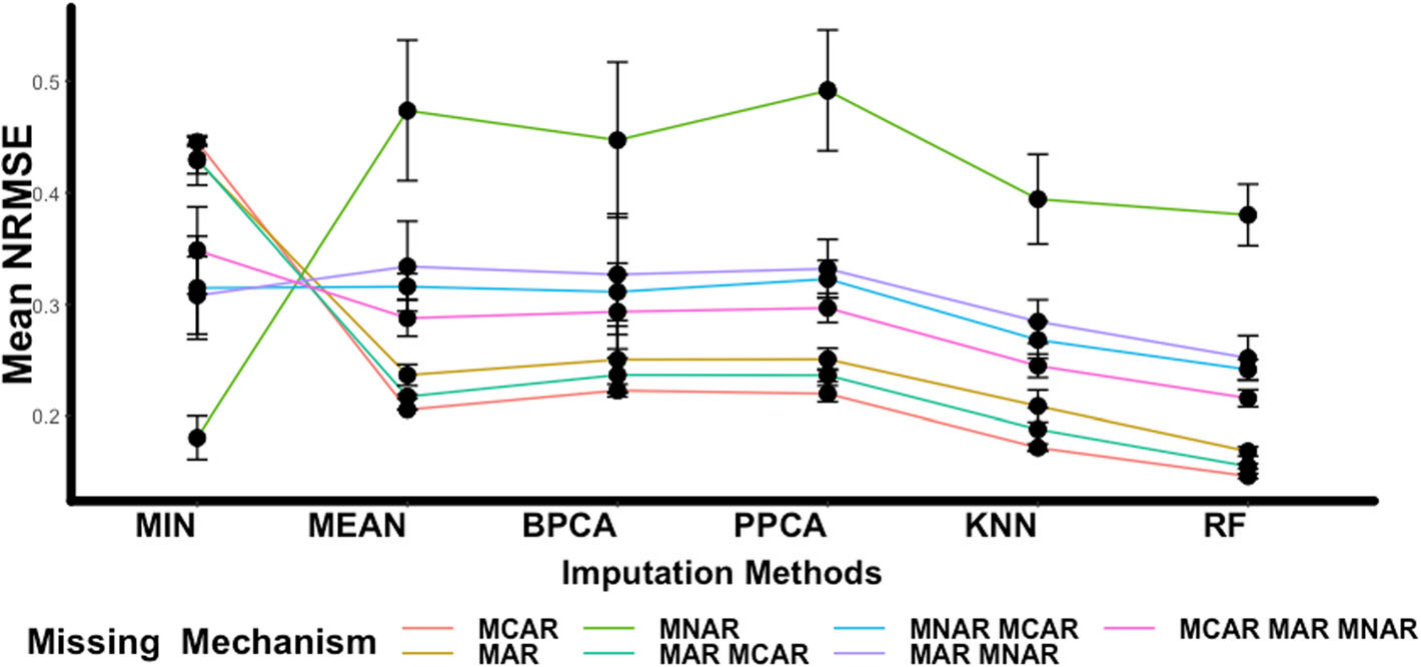
Summary Results. Summary results for six imputation methods; MIN, MEAN, BPCA, PPCA, KNN and RF. The general trend of the methods is being presented here with the y-axis being the average NRMSE for the four percentages of missing values (5, 10, 20 and 30%) together after 100 permutations. Each line shows one missing mechanisms (represented by a different color); MCAR, MAR, MNAR, MCAR-MAR, MCAR-MNAR, MAR-MNAR, MCAR-MAR-MNAR, and each black dot represents the average NRMSE with the error bars being the standard deviations of the NRMSEs for 100 permutations. The error bars are useful here because they report the uncertainty of the estimation of the imputed value per method

Figure 4 shows the summary results from six out of nine imputation methods that we evaluated; MIN, MEAN, BPCA, PPCA, KNN, and RF. This choice was based on the performances of the methods illustrated in the heatmaps in Fig. 3. For more details two summary tables (Tables S1–S2) containing the mean, standard deviation of the average NRMSEs of every imputation method for all the four percentages of missing values have been added in the Additional file 1. As suggested by these results, the most troublesome missingness is the MNAR with the MIN imputation is the best approach by having the smallest NRMSE. The rest of the five methods (MEAN, BPCA, PPCA, KNN, and RF) have high NRMSEs and also standard deviations. A relatively high error bar suggests that the method in question is not stable when imputing missing values and for each permutation provides different estimates. For the MCAR and MAR mechanism the conclusions are the same as in the heatmaps, with RF being the most appropriate choice. Furthermore, for the remaining four kinds of mixed missing mechanisms (MCAR-MAR, MCAR-MNAR, MAR-MNAR, and MCAR-MAR-MNAR) the performances are similar with MCAR and MAR cases, with RF being the best imputation method followed by KNN.

## Discussion

Here we tested nine imputation methods on seven different missing mechanisms with twelve metabolomics datasets. Our results indicated that RF was the most effective means for imputing metabolomics datasets based on the lowest NRMSE value and error bars in nearly all the simulated datasets representing different combinations of missing values. RF is a powerful method, even though the whole imputation procedure with RF has a computation expense due to the intensive iterations (Additional file 1: Figure S7), it is well known that it can handle both parametric and non-parametric data sets of complex linear and non-linear problems and there is no need to perform preprocessing at the data beforehand. On the other hand SVD where is a popular approach for data analysis and data processing (e.g., dimension reduction), PPCA which is basically PCA with an expectation– maximization (EM) approach and BPCA which is based on three processes, including principal component (PC) regression, Bayesian estimation, and an EM-like repetitive algorithm, they perform better if the data are being transformed first before imputation [2]. In our experimental setting we didn’t consider any preprocessing methods, even when this is common in other imputation studies, because in most metabolomics studies, almost half of the molecular features do not follow the normal distribution as their values are highly positively or negatively skewed. When a molecular feature is Log-transformed in order to meet the normality assumptions before imputing, we are not only changing the distribution of that particular molecular feature but also we disturb the relationships between that molecular feature with the rest of the data, and therefore, this approach can lead in imputing outliers or/and creating more bias than just imputing the skewed molecular feature.

One aspect of our results that was not aligned with the findings from another study [20], was the very poor performance of the SVD imputation method. SVD begins the imputation procedure by replacing all missing values with zero values and then iterates through singular value decompositions until convergence. This procedure may create effects on the data structure that can alter the factorization of the data matrices. In theory that shouldn’t happen because the errors in the prediction that are being introduced in the initialization step should be cancelled out by the matrix-factorization approach. On the other hand, BPCA and PPCA also rely on dimension reduction, performed better than SVD. This was because they include a probabilistic model that minimize the principal axes that are not relevant, and make these two methods more robust to changes in the data structure and in the cases of MCAR, MAR and MCAR -MAR.

We also observed that in the cases of MNAR, MCAR-MAR-MNAR, MAR-MNAR, MCAR-MNAR the performance of BPCA improved when the proportion of missing values increased and this phenomenon, which may seem at first contradictory, could be due to the fact that multivariate models perform better in a more uniformly distributed missingness across datasets.

The treatment of missing data by single value replacement, such as MIN, performs better when missing data arise from censoring below the detection limit, while other methods, such as RF, which is a method based on local structures, performs better when randomness is involved. KNN method performs slightly better than MEAN imputation which was expected since it is considered as an advancement over the MEAN imputation [17]. However, KNN showed instability in the prediction of missing values especially in the MAR case and this spurious performance is highlighting the fact that this method probably is not the most suitable for imputing such complex datasets.

In addition, it has been shown that certain methods favor more specific mechanisms than others, but there is no imputation method so far that works well for all three types of mechanisms [20]. By closely investigating the results presented in Figs. 3 and 4, it seems that RF is estimating missing values with the lowest prediction error. Its performance is consistent in the four percentages of missing values and for the majority of the missing mechanisms. The exception is the left-truncated MNAR, where the MIN performs better. These results coincide with other studies as well, that tested the performance of RF with other methods [2, 20].

However, in the case of the MNAR-MCAR-MAR, BPCA and MIN had similar performances with RF and KNN, especially for higher percentages of missing values of 20 and 30%. This result could indicate that as the number of missing values increases, the left truncation of missingness starts to affect the data while other kinds of missing mechanisms are also present. If the data are left truncated then the preferable option will be the MIN or any other imputation method, such as KNN-TN and GSimp, that perform well for this type of missingness as has been proposed also earlier [11, 20, 21].

In this work, the missing values were controlled and removed using one of the three earlier characterized missing mechanisms or the combination of those. However, most of the times in real life the reason for a value to be absent is unknown. In many studies different imputation strategies have been compared, or it has been investigated that how different imputation methods can alter the biological information within simulated or real metabolomics datasets [2, 16, 18]. The detection of the missing mechanism is not typically performed in metabolomics field [22] but some efforts have been done in other research areas focusing on simulated data only [13]. If the missing mechanism can be detected with high certainty, it could reduce the bias occurring from the improper choice of an imputation method.

One limitation that our study has, is in the way the current simulation process of missing values is carried out. We cannot fully control the missing patterns simulation as we indented. That means that even if intended to remove the data with the MAR mechanism we may create missing patterns by random chance that resemble the MNAR mechanism especially when the percentage of the missing values starts to increase. In order to reduce this effect, we use moderate sizes of missingness, no higher than 30%. Higher percentages regardless the missing mechanism tends to create MNAR missing patterns.

In this study, also we did not include any information about the different groupings inside the imputation study. When selecting the subset of 200 molecular features from the metabolomics data, we were focusing on data that was present for all the measured samples, in order to be able to simulate the missingness. We do acknowledge that when addressing complete metabolomics dataset, the grouping needs to be taken into account to avoid bias, and even introducing error, and therefore the imputation needs to be potentially performed in a group-wise manner. However, this would require further development to the utilized methods.

## Conclusion

Type and rate of missingness affects the performance and suitability of imputation methods. In conclusion, RF-based imputation method performs best in most of the tested scenarios, including combinations of different types and rates of missingness. Therefore, we recommend using RF-based imputation for imputing missing metabolomics data, since typically the origin of missingness is not known in advance. In addition, our approach to evaluate the performance of imputation methods on metabolomics datasets is applicable also to other high-dimensional data that contain missing values.

## Methods

### Metabolomics data

The metabolomics datasets used in the current study were obtained from two human based-nutritional studies carried out at the University of Eastern Finland. The Kuopio Ischaemic Heart Disease Risk Factor Study (KIHD) is an epidemiological study focusing on the effect of diet and lifestyle on cardiovascular disease risk in middle-aged men from eastern Finland [23]. A subset of 258 participants was randomly selected for a study focusing on the metabolic impact of egg consumption [24].

(https://www.uef.fi/web/nutritionepidemiologists/kuopio-ischaemic-heart-disease-risk-factor-stud-kihd-1984-).

The other dataset used in the current work was obtained from a human dietary intervention, namely the Berry Study (BS) [25, 26]. BS was a controlled 16-week interventional trial, where plasma was collected from 47 individuals in two time periods; at the baseline (start of intervention) and at the 8-week follow up (end of intervention). Twelve individuals served as a control group, and the rest were divided into two experimental groups consuming two different types of berries.

The samples from these two studies were analyzed by UHPLC-qTOF-MS system (Agilent Technologies, Waldbronn, Karlsruhe, Germany) that consisted of a 1290 LC system, a Jetstream electrospray ionization (ESI) source, and a 6540 UHD accurate-mass qTOF spectrometer. The samples were analyzed using two different chromatographic modes, i.e. reversed phase (RP) and hydrophilic interaction (hilic) chromatography and the data were acquired in both positive (+) and negative (−) polarity. Here we consider each of these four analytical modes as separate datasets. The data pre-processing was carried out as described earlier [25, 27].

From the KIHD study we created four datasets (four analytical modes) and from the BS study, eight datasets representing the two time points and four analytical modes (Fig. 1). In order to create the datasets for the evaluation of the imputation methods, we considered molecular features that were present in all samples. Table 1 provides information about how many molecular features we had in each datasets and the total number of missing values. During the simulation processes, every time we repeated the simulation, we randomly picked one dataset out of the 12, and randomly selected 200 molecular features from this particular dataset that does not include any missing values, and this new sub-dataset was used to simulate missing values and test the imputation methods thereafter. Additional boxplots and correlation plots show the new complete sub-datasets in more detailed (Additional file 1: Figures S1-S6).

**Table 1:** Information about the total number of molecular features, number of missing values and total number of complete molecular features in each dataset

Study	LC-MS analytical mode	Total number of molecular features	Number of missing values per dataset	Number of molecular features without missing values
KIHD	Hillic positive	2407	7519	204
	Hillic negative	1228	64,256	299
	RP positive	1228	24,451	509
	RP negative	2407	95,079	498
BS				
Start(T_0_)	Hillic positive	1290	7140	518
End (T_1_)	Hillic positive	1290	7131	508
Start(T_0_)	Hillic negative	725	2701	383
End (T_1_)	Hillic negative	725	2766	383
Start(T_0_)	RP positive	1177	11,679	1066
End (T_1_)	RP positive	1177	11,526	1152
Start(T_0_)	RP negative	2286	4643	578
End (T_1_)	RP negative	2286	5146	559

### Missing mechanisms simulation

MCAR missingness was simulated by randomly removing values from the data set using the uniform distribution. Different rates of missingness were simulated by removing different proportions of the values (5, 10, 20, and 30%).

In the MAR missing mechanism, the missing values depend on the observed part of the data. In our simulation, we model a situation where a high abundance of a molecular feature X_1_ will lead to missingness of a molecular feature X_2_ in the same sample. The simulation process begins by randomly choosing two different molecular features; X_1_ and X_2_. We sort the values of X_1_ from minimum to maximum, and choose a cut-off percentage point randomly from chi-squared distribution, divide it by 30 and limit to range 0..1. This approach will limit the cut-off percentage between 0 to 100%, with mean of 3.3% and standard deviation of 4.7% (empirically simulated with 1 million values). This selected cut-off percentage will be used to set this proportion of highest X_2_ values to missing, simulating MAR missingness. After this, new X_1_ and X_2_ are randomly selected, and the procedure is repeated until total desired proportion of total missingness (5, 10, 20%, or 30%) for the whole data set is reached.

The MNAR missing mechanism is very similar to the MAR simulation process. We chose randomly one molecular feature, X_1_ and sort its values from minimum to maximum. Then we repeat the same process as in the MAR case with the only difference here being that the missing values will be created on X_1,_ and it will be below the cut point if we choose left truncation or above the cut point if we choose right truncation, which in this workflow the right truncation wasn’t used. This process is repeated until we reach the desirable proportion of missing values (5, 10, 20, and 30%).

In the cases of the mixed missingness e.g. MCAR-MAR-MNAR, the simulation process was done in three steps. If for example we wanted to remove 30% of the data using this mechanism, in the first step 10% of the data will be removed using the MCAR method, then the resulting output data will be passed to the MAR mechanism to remove another 10% and in the end the output data will be passed to the MNAR mechanism in order to remove the last 10%. Always in the mixed missingness mechanisms the missing percentages are equally distributed among MNAR, MAR and MCAR. The reasoning behind this choice was to automatize the whole simulated procedure.

All the functions and code for the simulation process are available on GitHub: (website: https://github.com/mariekok/impute-metabolomics). Furthermore, the function that is used to simulate the three different missing mechanisms is called simulate_missingness() and it can also been found in the Additional file 1.

**Fig. 5:**
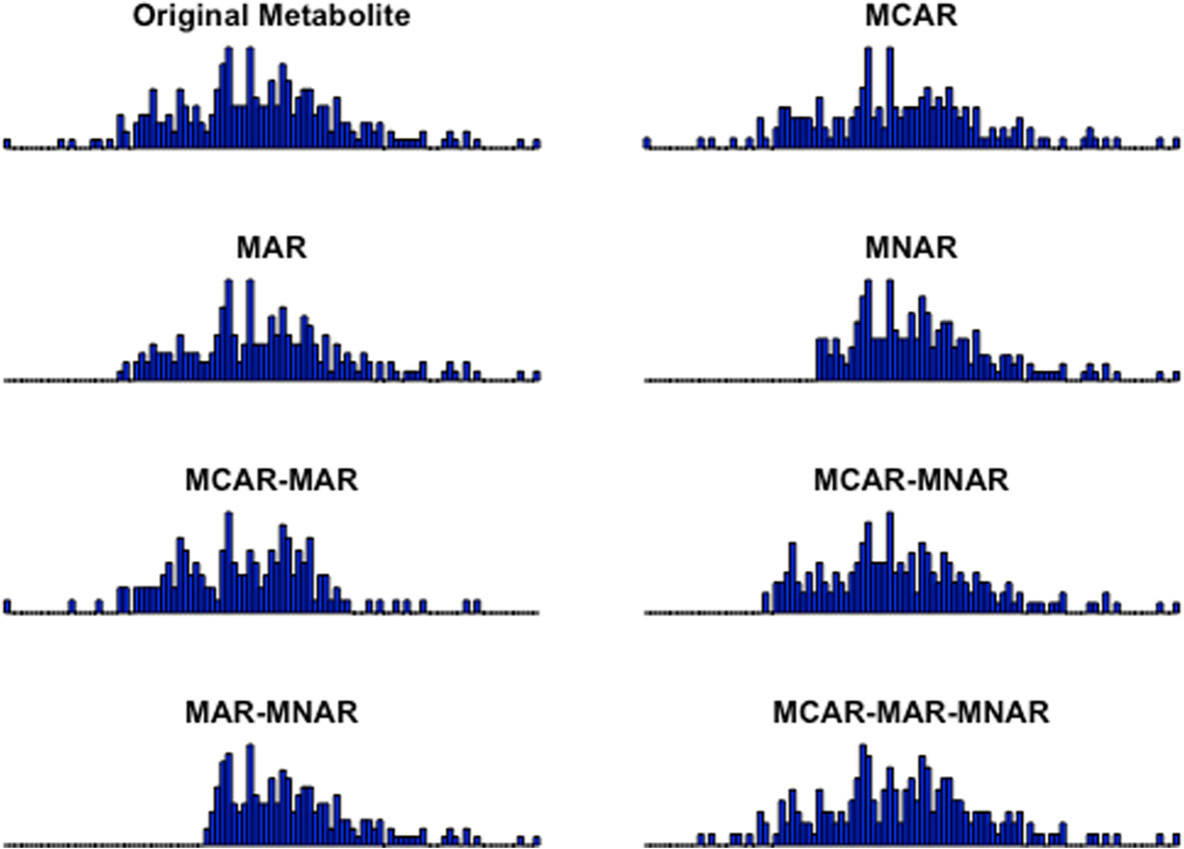
Distribution based examples of the simulated missing mechanisms. Simulation of missing values for seven different missing mechanisms; Missing Completely At Random (MCAR), Missing At Random (MAR), Missing Not At Random (MNAR), MCAR-MAR, MCAR-MNAR, MNAR-MAR, MNAR-MCAR-MAR for a molecular feature X. The range of missing values is set to 30%

Figure 5 illustrates how missing values in seven different missing mechanisms (MCAR, MAR, MNAR, MCAR-MAR, MCAR-MNAR, MAR-MNAR, MCAR-MAR-MNAR) affect the distribution of a molecular feature, as exemplified with the case of having 30% of missing values in the whole dataset.

### Imputation methods

We compared nine imputation methods that have been suggested earlier for metabolomics data-analysis. The methods were divided into three main categories. The first category is the imputation by a single value replacement and includes the mean imputation (MEAN), the minimum observed value, ½ minimum (½ MIN) observed value and zero (ZERO) imputation. The second category is the imputation methods based on local structures and that includes random forest (RF) [28, 29], and k- Nearest Neighbors (KNN) [30–33].The last category contains the imputation methods based on global structures like Singular Value Decomposition (SVD) [34], Probabilistic Principal Component Analysis (PPCA) [7, 13, 35, 36] and Bayesian Principal Component Analysis (BPCA) [36–38]. The comparison of imputation methods was done by using Normalized Root Mean Square Error (NRMSE), which has been also earlier suggested for similar purpose [20, 39]. NRMSE calculates the difference in the estimation between the imputed value and the original value.

$NRMSE=\sqrt{\frac{mean\left({\left({X}^{comp}-{X}^{imp}\right)}^2\right)}{\mathit{\operatorname{var}}\left({X}^{comp}\right)}}$, where *X*^*comp*^ is a dataset without missing values and *X*^*imp*^ is the same dataset with the missing values being imputed.

### Imputation study workflow

We tested different imputation strategies in real datasets from untargeted LC-MS studies. We simulated MNAR, MCAR, MAR missingness and the different combinations of missingness, namely MCAR-MAR, MCAR-MNAR, MNAR-MAR, and MNAR-MCAR-MAR**,** for the evaluation of nine imputation methods (ZERO, MEAN, MIN, ½ MIN, SVD, PPCA, BPCA, RF, and KNN) in four different proportions of missing values; 5, 10, 20, and 30%. The whole process was repeated 100 times. In each permutation, one dataset out of the 12 was randomly chosen and 200 new molecular features without any missing values were randomly selected. To this sub-dataset every one of the seven different missing mechanisms were simulated one at a time and with four different percentages of missing values. The new datasets with simulated missing values were used to evaluate the performance of nine imputation methods. The performance of these methods was estimated by calculating NRMSE between the imputed new datasets and the “complete” dataset. A detailed description of the workflow can be seen in Fig. 2.

## Supplementary information

**Additional file 1.** Contains descriptive statistics measures such as boxplots and correlation plots for the simulated sub-datasets, two summary tables that illustrate the average NRMSEs for all four proportion of missing values for every imputation method and for every type of missingness and one plot illustrating the computational times for every imputation method in every proposition of missing values.

## Data Availability

The algorithms were all coded in the R language, version 3.3.1 with the packages: foreach, dplyr, futile.logger, missForest, pcaMethods, doMC, speedglm, imputeLCMD, ggplot2, reshape2, tidyr, matrixStats, VIM. The R code for imputation functions and R scripts are available in GitHub (https://github.com/juuussi/impute-metabo). R itself and all packages used are available from CRAN at (http://CRAN.R-project.org/). The data from the KIHD and BS studies are not publicly available due to sensitive personal information of the study subjects.

## Abbreviations

½ MIN: Half minimum value
BPCA: Bayesian Principal Component Analysis
BS: Berry Study
KIHD: Kuopio Ischemic Heart Disease Risk Factor Study
KNN: K-Nearest Neighbors
MAR: Missing At Random
MCAR: Missing At Random
MIN: Minimum value
MNAR: Missing Not At Random
PPCA: Probabilistic Principal Component Analysis
RF: Random Forest
SVD: Singular Value Decomposition
