# Supplementary material for: Random forest-based imputation outperforms other methods for imputing LC-MS metabolomics data: a comparative study
Source: BMC Bioinformatics. 2019 Oct 11;20:492. doi: 10.1186/s12859-019-3110-0 (PMC6788053; doi:10.1186/s12859-019-3110-0)
Supplement: Supplementary file 1 — Additional file 1. Contains descriptive statistics measures such as boxplots and correlation plots for the simulated sub-datasets, two summary tables that illustrate the average NRMSEs for all four proportion of missing values for every imputation method and for every type of missingness and one plot illustrating the computational times for every imputation method in every proposition of missing values. [file 12859_2019_3110_MOESM1_ESM.docx]

**Appendix 1. Descriptive Statistics**

Boxplots and Pairwise correlation plots (Figures 1-6) for 200 randomly and without missing values molecular features for every dataset , 1:Hillic negative,2:Hillic positive,3: RP negative,4: RP positive mode, 5:Hillic Negative time :start, 6:Hillic negative time :end,7:Hillic positive time: start,9 :RP negative time :start,10:RP negative time :end,11:RP positive time: start, 12:RP positive time: end.


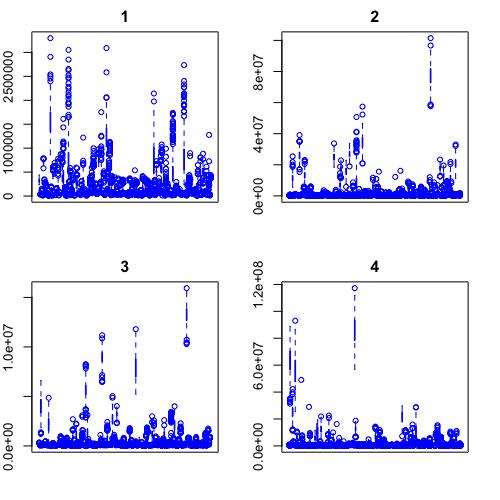


*Figure S1. Boxplots for 200 complete random molecular features chosen from the KHID datasets,1:Hillic negative,2:Hillic positive,3: RP*

*negative,4: RP positive mode. The X axis represent each molecular feature and the Y axis the raw intensities.*


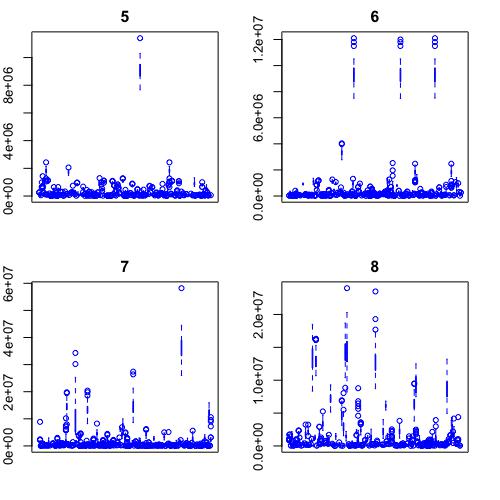


*Figure S2. Boxplots for 200 complete random molecular features chosen from the BS datasets,5:Hillic Negative time :start,6:Hillic negative time :end,7:Hillic positive time: start, 8:Hillic positive time: end. The X axis represent each molecular feature and the Y axis the raw intensities.*


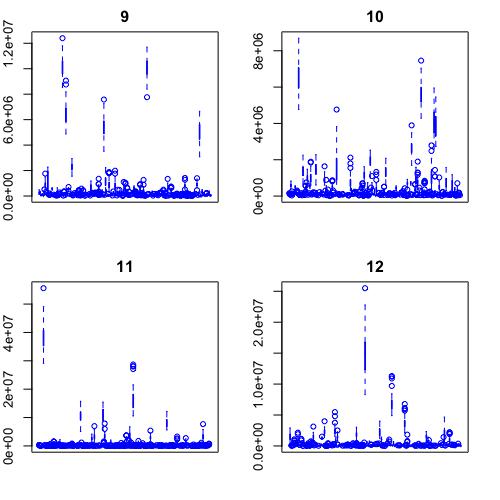


*Figure S3. Boxplots for 200 complete random molecular features chosen from the BS datasets,9 :RP negative time :start,10:RP negative time :end,11:RP positive time: start, 12:RP positive time: end. The X axis represent each molecular feature and the Y axis the raw intensities.*


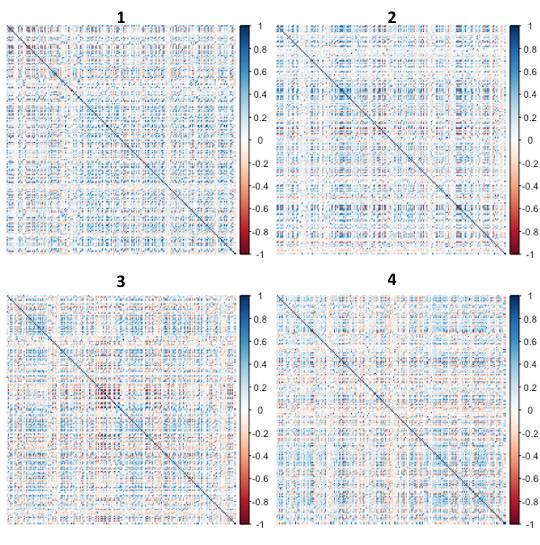


*Figure S4.Pairwise correlations from the 200 complete and randomly selected molecular features from KIHD datasets,1:Hillic negative,2:Hillic positive,3: RP negative,4: RP positive mode*


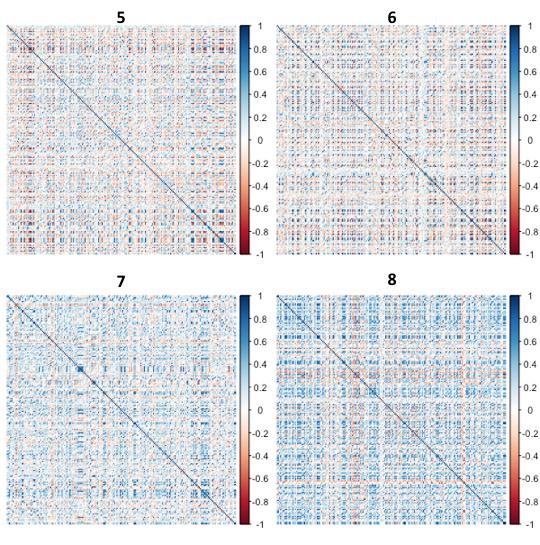


*Figure S5.Pairwise correlations from the 200 complete and randomly selected molecular features chosen from the BS datasets,5:Hillic Negative time :start,6: Hillic negative time :end,7:Hillic positive time: start, 8:Hillic positive time: end*


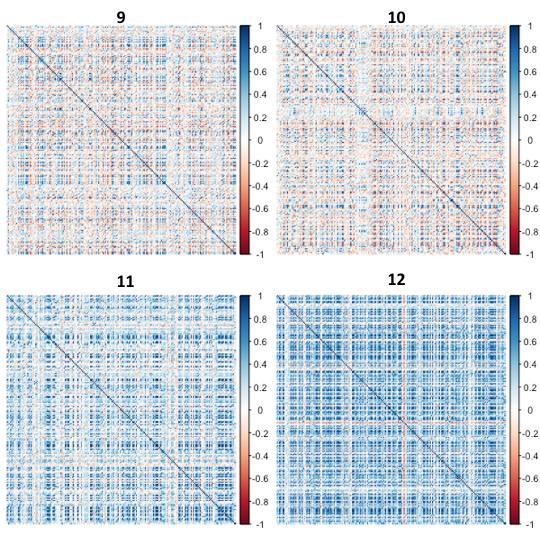


*Figure S6.Pairwise correlations from the 200 complete and randomly selected molecular features chosen from the BS datasets,9:RP negative time :start,10:RP negative time :end,11:RP positive time: start, 12:RP positive time: end*


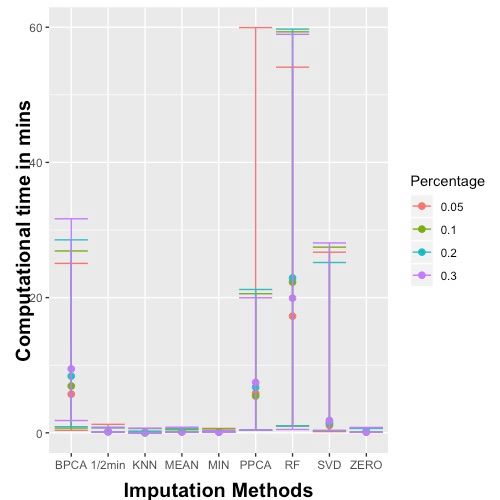


*Figure S7.Boxplot of the nine imputation methods versus the total computational time per missing percent*

For better understanding of the simulation procedure all the code can be found on GitHub: (website: <https://github.com/mariekok/impute-metabolomics>)

Here we present the function that we created to simulate the three main types of missingness (MCAR,MNAR,MAR)

#' Title simulate_missingness

#' Uses the simulated data to create the different types of missingness (MCAR,MNAR,MAR)

#' using different percentages of missingness

#' @param data matrix with simulated data

#' @param mcar percentage of missingness in Missing Completely at Random

#' @param mar percentage of missingness in Missing at Random

#' @param mnar percenatge of missingness Missing not at Random

#' @param mnar.type type of truncation 'left' or right

#'

#' @return simulated_data

#' @export

#'

#' @examples miss_data <- simulate_missingness(data=simulated_data, mcar=0.01)

#'####################################################

simulate_missingness <- function(data, mcar=0, mar=0, mnar=0, mnar.type="left", mar.type="left") {

if(class(data) != "matrix") {

stop("Variable data should be a matrix.")

}

if ( ((mcar + mar + mnar) > 1) || ((mcar + mar + mnar) < 0)) {

stop("Sum of mcar, mar and mnar should be between 0 and 1.")

}

simulated_data <- data

if (mcar > 0){

mcar_distribution = runif(nrow(data)*ncol(data), min=0, max=1)

simulated_data = matrix(ifelse(mcar_distribution<mcar, NA, data), nrow=nrow(data), ncol=ncol(data))

}

if (mnar > 0) {

added_mnar <- 0

initial_nas <- sum(colSums(is.na(simulated_data)))

current_nas <- initial_nas

simulated_data_n <- ncol(simulated_data) * nrow(simulated_data)

# condition of proportion of missigness

while (((current_nas - initial_nas) / simulated_data_n) < mnar) {

# Select random variable (molecular feature X1)

variable_index <- sample(1:ncol(simulated_data), 1)

# What percentage of variable to set missing

cut_percentage <- 0

while (cut_percentage <= 0) {

cut_percentage <- rchisq(1, df=1) / 30

}

cut_percentage <- min(cut_percentage, 1)

# How many values to set missing

cut_index <- floor(cut_percentage * nrow(simulated_data))

sorted_variable <- sort(simulated_data[,variable_index])

# Set values to missing

if (mnar.type == "right") {

# Corresponding cut-off point for values

cut_point <- sorted_variable[length(sorted_variable) - cut_index]

simulated_data[simulated_data[,variable_index] > cut_point, variable_index] <- NA

} else {

# Corresponding cut-off point for values

cut_point <- sorted_variable[cut_index]

simulated_data[simulated_data[,variable_index] < cut_point, variable_index] <- NA

}

# Counter to check how much MNAR missingness has been added to data

current_nas <- sum(colSums(is.na(simulated_data)))

}

}

if (mar > 0) {

added_mar <- 0

initial_nas <- sum(colSums(is.na(simulated_data)))

current_nas <- initial_nas

simulated_data_n <- ncol(simulated_data) * nrow(simulated_data)

# condition of porpotion of missigness

while (((current_nas - initial_nas) / simulated_data_n) < mar) {

# Select random variable (molecular feature X1)

variable_index <- sample(1:ncol(simulated_data), 1)

# Select random variable (molecular feature X2 different than X1)

variable_index2 <- sample(setdiff(1:ncol(simulated_data), variable_index), 1)

# What percentage of variable to set missing

cut_percentage <- 0

while (cut_percentage <= 0) {

# select a random percentage of missingness from chisquared distribution (mean missigness ~3.3%)

cut_percentage <- rchisq(1, df=1) / 30

}

cut_percentage <- min(cut_percentage, 1)

# How many values to set missing

cut_index <- floor(cut_percentage * nrow(simulated_data))

sorted_variable <- sort(simulated_data[,variable_index])

# Set values to missing

if (mar.type == "right") {

# Corresponding cut-off point for values

cut_point <- sorted_variable[length(sorted_variable) - cut_index]

simulated_data[simulated_data[,variable_index] > cut_point, variable_index2] <- NA

} else {

# Corresponding cut-off point for values

cut_point <- sorted_variable[cut_index]

simulated_data[simulated_data[,variable_index] < cut_point, variable_index2] <- NA

}

# Counter to check how much MNAR missingness has been added to data

current_nas <- sum(colSums(is.na(simulated_data)))

}

}

simulated_data

}

*Table S1. Summary statistics for the average performance of nine imputation method in total for the MCAR ,MAR and MNAR types of missingness for all four percentages of missing values (5%,10%,20%,30%)*

| **Methods** |  | **MCAR** | |  |  | **MAR** | |  | **MNAR** | |
| --- | --- | --- | --- | --- | --- | --- | --- | --- | --- | --- |
|  | ***Mean*** | ***SD*** | ***95%CI*** | ***Mean*** | ***SD*** |  | ***95%CI*** | ***Mean*** | ***SD*** | ***95%CI*** |
| **zero** | 1.060 | 0.0 | [1.06,1.06] | 1.067 | 0.004 |  | [1.06677, 1.06723] | 1.063 | 0.005 | [1.06272, 1.06328] |
| **1/2min** | 0.736 | 0.003 | [0.71903, 0.75297] | 0.726 | 0.006 |  | [0.72566, 0.72634] | 0.488 | 0.007 | [0.4876, 0.4884] |
| **min** | 0.446 | 0.005 | [0.44572, 0.44628] | 0.429 | 0.01 |  | [0.42843, 0.42957] | **0.181** | 0.024 | [0.17964, 0.18236] |
| **mean** | 0.206 | 0.004 | [0.20577, 0.20623] | 0.237 | 0.008 |  | [0.23655, 0.23745]] | 0.474 | 0.073 | [0.46987, 0.47813] |
| **SVD** | 0.924 | 0.063 | [0.92044, 0.92756] | 1.112 | 3.646 |  | [0.90571, 1.31829] | 1.402 | 3.921 | [1.18015, 1.62385] |
| **BPCA** | 0.223 | 0.026 | [0.22253, 0.22547] | 0.251 | 0.054 |  | [0.24794, 0.25406] | 0.447 | 0.14 | [0.43908, 0.45492] |
| **PPCA** | 0.22 | 0.007 | [0.2197, 0.2203] | 0.251 | 0.042 |  | [0.24876, 0.25324] | 0.492 | 0.157 | [0.48166, 0.50234] |
| **KNN** | 0.172 | 0.005 | [0.17172, 0.17228] | 0.209 | 0.008 |  | [0.20855, 0.20945] | 0.394 | 0.079 | [0.3888, 0.3992] |
| **RF** | **0.146** | 0.004 | [0.14578, 0.14622] | **0.169** | 0.01 |  | [0.16844, 0.16956] | 0.38 | 0.052 | [0.377, 0.383] |

*Table S2. Summary statistics for the average performance of nine imputation method in total for the MNAR-MAR, MAR-MCAR,MNAR-MCAR,MNAR-MCAR-MAR types of missingness for all four percentages of missing values (5%,10%,20%,30%)*

| **Methods** |  | **MNAR MAR** | |  | **MAR MCAR** | |  | **MNAR MCAR** | |  | **MNAR MAR MCAR** | |
| --- | --- | --- | --- | --- | --- | --- | --- | --- | --- | --- | --- | --- |
|  | ***Mean*** | ***SD*** | ***95%CI*** | ***Mean*** | ***SD*** | ***95%CI*** | ***Mean*** | ***SD*** | ***95%CI*** | ***Mean*** | ***SD*** | ***95%CI*** |
| **zero** | 1.063 | 0.007 | [1.05904, 1.06696] | 1.062 | 0.003 | [1.06183, 1.06217] | 1.058 | 0.001 | [1.05794, 1.05806] | 1.060 | 0.003 | [1.06183, 1.06217] |
| **1/2min** | 0.627 | 0.018 | [0.62598, 0.62802] | 0.727 | 0.005 | [0.72672, 0.72728] | 0.63 | 0.014 | [0.6292, 0.6308] | 0.66 | 0.011 | [0.6594, 0.6606] |
| **min** | 0.308 | 0.028 | [0.30642, 0.30958] | 0.43 | 0.008 | [0.4295, 0.4305] | 0.315 | 0.023 | [0.3137, 0.3163] | 0.349 | 0.02 | [0.34787, 0.35013] |
| **mean** | 0.334 | 0.056 | [0.33083, 0.33717] | 0.217 | 0.005 | [0.21672, 0.21728] | 0.316 | 0.022 | [0.31476, 0.31724] | 0.288 | 0.023 | [0.2867, 0.2893] |
| **SVD** | 1.012 | 0.346 | [0.99242, 1.03158] | 0.96 | 0.251 | [0.9458, 0.9742] | 1.103 | 1.784 | [1.00206, 1.20394] | 1.062 | 2.223 | [0.93622, 1.18778] |
| **BPCA** | 0.327 | 0.087 | [0.32208, 0.33192] | 0.237 | 0.037 | [0.23491, 0.23909] | 0.311 | 0.076 | [0.3067, 0.3153] | 0.294 | 0.066 | [0.29027, 0.29773] |
| **PPCA** | 0.332 | 0.071 | [0.32792, 0.33608] | 0.236 | 0.023 | [0.23474, 0.23726] | 0.323 | 0.043 | [0.32037, 0.32563] | 0.297 | 0.042 | [0.29457, 0.29943] |
| **KNN** | 0.285 | 0.039 | [0.28279, 0.28721] | 0.188 | 0.003 | [0.18783, 0.18817] | 0.268 | 0.014 | [0.26721, 0.26879] | 0.245 | 0.021 | [0.24378, 0.24622] |
| **RF** | **0.252** | 0.038 | [0.24985, 0.25415] | **0.155** | 0.005 | [0.15472, 0.15528] | **0.242** | 0.015 | [0.24115, 0.24285] | **0.216** | 0.021 | [0.21481, 0.21719] |

cccc
